# Magnetoimpedance Effect in the Ribbon-Based Patterned Soft Ferromagnetic Meander-Shaped Elements for Sensor Application

**DOI:** 10.3390/s19112468

**Published:** 2019-05-29

**Authors:** Zhen Yang, Anna A. Chlenova, Elizaveta V. Golubeva, Stanislav O. Volchkov, Pengfei Guo, Sergei V. Shcherbinin, Galina V. Kurlyandskaya

**Affiliations:** 1 Department of Magnetism and Magnetic Nanomaterials, Ural Federal University, 620002 Ekaterinburg, Russia; zhc025@alumni.sjtu.edu.cn (Z.Y.); anniaally@gmail.com (A.A.C.); golubeva.elizaveta.v@gmail.com (E.V.G.); stanislav.volchkov@urfu.ru (S.O.V.); scher30@yandex.ru (S.V.S.); 2 School of Physics and Electronic Engineering, Xinyang Normal University, Xinyang 464000, China; guopengfei2010@126.com; 3 Institute of Metal Physics UD RAS, 620016 Ekaterinburg, Russia; 4 Institute of Electrophysics UD RAS, 620016 Ekaterinburg, Russia; 5 University of the Basque Country UPV-EHU, 48940 Leioa, Spain

**Keywords:** Magnetoimpedance effect, amorphous ribbons, patterned ribbons, meander sensitive element, magnetic field sensor

## Abstract

Amorphous and nanocrystalline soft magnetic materials have attracted much attention in the area of sensor applications. In this work, the magnetoimpedance (MI) effect of patterned soft ferromagnetic meander-shaped sensor elements has been investigated. They were fabricated starting from the cobalt-based amorphous ribbon using the lithography technique and chemical etching. Three-turn (S1: spacing s = 50 μm, width w = 300 μm, length l = 5 mm; S2: spacing s = 50 μm, width w = 400 μm, length l = 5 mm) and six-turn (S3: s = 40 μm, w = 250 μm, length l = 5 mm; S4: s = 40 μm, w = 250 μm and l = 8 mm) meanders were designed. The ‘n’ shaped meander part was denominated as “one turn”. The S4 meander possesses a maximum MI ratio calculated for the total impedance ΔZ/Z ≈ 250% with a sensitivity of about 36%/Oe (for the frequency of about 45 MHz), and an MI ratio calculated for the real part of the total impedance ΔR/R ≈ 250% with the sensitivity of about 32%/Oe (for the frequency of 50 MHz). Chemical etching and the length of the samples had a strong impact on the surface magnetic properties and the magnetoimpedance. A comparative analysis of the surface magnetic properties obtained by the magneto-optical Kerr technique and MI data shows that the designed ferromagnetic meander-shaped sensor elements can be recommended for high frequency sensor applications focused on the large drop analysis. Here we understand a single large drop as the water-based sample to analyze, placed onto the surface of the MI sensor element either by microsyringe (volue range 0.5–500 μL) or automatic dispenser (volume range 0.1–50 mL).

## 1. Introduction

The magnetoimpedance (MI) effect is a classic electromagnetic phenomenon which can be described as a significant change of the total impedance of a ferromagnetic conducting sensor element applied to an external magnetic field when a high frequency alternating current flows through it [1,2,3]. The first magnetic field sensors working on the principle of the change of alternating current resistance applied to an external magnetic field were reported by Makhotkin et al. [4] even before the MI phenomenon was given its proper name [2,3].

In recent years, MI sensors have attracted special attention for their promising technological applications in different fields [5,6,7,8]. Cobalt-based amorphous ribbons show good MI characteristics with high sensitivity with respect to applied magnetic fields, sufficient even for biosensor applications [9,10]. Cobalt-based rapidly quenched materials have reasonably high saturation magnetization and nearly zero magnetostriction constant value, characteristics that make them good candidates for very different applications [11,12,13]. One of the shortcomings of the rapidly quenched ribbon-based materials is their relatively large size. It was shown previously that the shape anisotropy plays a crucial role and the MI ratio decreases significantly in the ribbons with a length of less than a few centimeters [14,15]. One of the solutions to such a problem was to create patterned elements in the shape of the meander [16,17,18] when the flat surface of the sensor element is required. Although meander geometry was previously applied with success for the case of MI multilayered structures [19,20,21], the proposed solution was shown to be useful in selected applications as the ribbon patterning technology is cheaper in comparison with the thin films case, despite the disadvantage of a lower degree of compatibility with semiconductor electronics, and the interaction effect resulting in a decrease of the overall sensitivity. The effective length in a meander-shaped MI sensor element is increased in comparison with the overall maximum dimension, providing the MI meander element a special advantage. At the same time, possible interactions between the fields created by the different parts of a meander can be complicated, and this geometry requires additional investigation.

Low working frequency is usually preferred for sensor applications as it makes the electronics circuit cheaper and insures easier processing of the electronic signals. At the same time, the frequency of the exciting current is an important parameter for determining the MI value [1,12]. Further development of electronic circuits and measurement techniques have possibly extended the frequency range of MI-based sensor applications [22,23,24] to higher frequencies where the signal-to-noise ratio can be improved [12].

Despite the fact that MI materials with a very high MI ratio and sensitivity, combined with a small size, have already been reported [25,26], there are particular biomedical applications for which flat biosensors are required to measure liquids with a relatively large bioanalyte drop. Such applications can be found in the environmental control and technological control of reservoirs. It is worth mentioning the existence of similar solutions found earlier for giant magnetoresistance-based elements for magnetic label detection [27]. In these cases, the resulting sensor elements consisted of wound lines forming the spiral structures. They were assembled into the element consisting of tens of the elements incorporated into the area of few squared millimeters. The signal for detection was collected from a number of elements after appropriate biochemical treatment. Therefore, we proposed to design a relatively large sensor element for the evaluation of the signal from one element. In future it might be useful for testing the composition with four elements in a bridge configuration.

In this work, the magnetic properties and magnetoimpedance of patterned Co-based amorphous ribbons were comparatively analyzed. We made the first step to work out the protocol of evaluation of magnetic properties of such complex subjects. MI was measured in the extended frequency range of 1–200 MHz. The patterning was done by lithography and chemical etching in the shape of meander sensor elements with three- or six-turns, with design focused on large drop analysis.

## 2. Materials and Methods

### 2.1. Fabrication of Ribbon-Based Patterned Soft Ferromagnetic Multi-Turn Sensitive Elements

First of all, we designed and developed small sensor elements in the geometry of the meander shape close to a square, aiming to develop a particular type of detector focused on the single large drop analysis of bioanalytes. Water droplet size is very important for different areas of analysis. Here, we understand a single large drop as the water-based sample to analyze, laced onto the surface of the MI sensor element either by microsyringe (volue range 0.5–500 μL) or automatic dispenser (volume range 0.1–50 mL). The fabrication process of patterned ribbons constitutes a series of steps. Initially the as-cast ribbon was produced by the rapid quenching technique [24,25] followed by cutting, field annealing, mechanical polishing and micromachining. Amorphous ribbons in the as-cast state were obtained with Co_75_Fe_10_Ni_2_Si_8_B_5_ nominal composition and a thickness of about 20 μm. Afterward, ribbons were cut in 4 × 3 cm pieces via the SPF-7100 die sawing system, and the subjected to transverse magnetic field annealing treatment in order to induce transverse magnetic anisotropy. The magnetic field during field annealing was applied perpendicular to the ribbon axis (perpendicular to the direction of the ribbon movement during the solidification). The details of the annealing process can be found elsewhere [23]. Mechanical polishing was performed by diamond sand-paper with a gradual reduction of the grain size in order to reduce the surface roughness having a negative impact on the value of MI sensitivity [28]. After polishing, the thickness of the ribbons was close to 15 μm. In order to reduce the etching error, chemical etching was carried out in a thermostatic water bath. Micromachining was done by micro electro-mechanical system (MEMS) technology, including bonding, lithography, and chemical etching. After etching, all patterned ribbons in the meander shape were polished again to ensure a smooth surface. The last request is especially important in biosensor applications for magnetic label detection.

**Figure 1:**
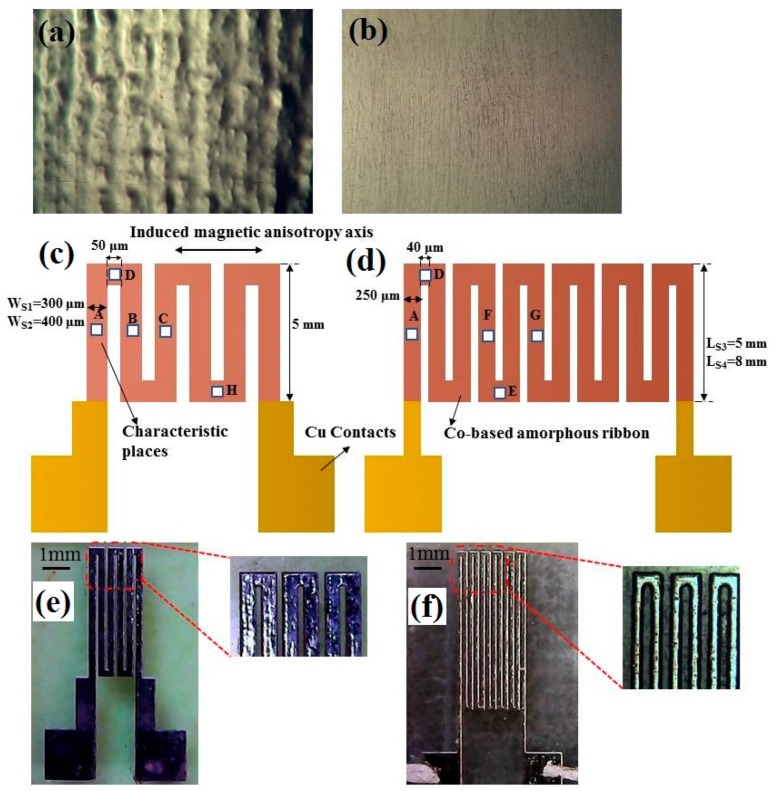
Surface micrograph of the Co_75_Fe_10_Ni_2_Si_8_B_5_ ribbon after chemical etching (**a**). Surface micrograph of the chemical etched Co_75_Fe_10_Ni_2_Si_8_B_5_ ribbon after polishing (**b**). The description of the selected types of the patterned MI meanders (**c**,**d**). General view of the fabricated patterned MI meanders S1 and S4 (**e**,**f**). Part (**c**) shows three-turn and part (**d**) shows six-turn meanders.

Although different geometries obtained by the employed technique were tested, we selected just a few of them with reasonable quality (Figure 1). With the amorphous ribbons it is impossible to ensure a flat cross-section of the samples for the individual line width below 0.1 mm. Planning the creation of the magnetic marker sensor for the in-liquid detection, one must keep the surface geometry as flat as possible. Meanders with a larger number of turns are difficult to fabricate by etching; therefore we selected some simple options for testing. Figure 1a,b shows the surface features of the amorphous ribbon after both chemical etching and polishing.

The micropatterned ribbon sensor elements were designed as compact n-shaped meander structures. Figure 1c,d shows selected examples of the meander geometry. The ‘n’ shaped meander part was denominated as a “one turn” sample.

Three-turn meanders were denominated as S1 and S2, and six-turn meanders were denominated as S3 and S4. Details of the geometries of all samples are given in Table 1. The length close to 8 mm was previously described in the literature as a critical length for non-patterned amorphous ribbon [13,14]. The spacing of 50 μm and length of 5 mm were used for S1 and S2 meanders and the same spacing of 40 μm and width of 250 μm were used for S3 and S4 meanders. Figure 1e,f shows a general view of the fabricated S1 and S4 meanders. High magnification allows an estimation of the quality of the fabricated sensitive elements.

### 2.2. Measurement of Surface Magnetic Properties of the Samples

Surface magnetic properties of the samples were investigated by the magneto-optical Kerr effect (MOKE) using a Kerr microscope (Evico magnetics GmbH, Dresden, Germany). MOKE measurements were performed by collecting the signal in the longitudinal mode, i.e., the component of magnetization lying in the sample plane and parallel to the direction of incidence of light was analyzed. Figure 1c, d shows some selected positions—A, B, C, D, E, F, G, H for MOKE measurement (white squares) according to the symmetric structure of the meander patterning. The signal was obtained from the plot area of 0.5 × 0.5 mm^2^. Some characteristic positions named by the same letter were picked up for the same location for different samples. The magnetic field up to 270 Oe was applied along the ribbon axis coinciding with longer part of the “n”- shaped direction. Meanwhile the magnetic field was also applied perpendicular to the ribbon axis for comparative analysis.

MOKE is a surface sensitive method that probes the surface magnetization. The origin of the high frequency alternating current impedance is connected to the change of the dynamic magnetic permeability and skin effect [1,2,3]. As the high frequency alternating current flows close to the surface, it is the surface anisotropy that makes a significant contribution to the MI effect value.

We made the first step to work out the protocol of evaluation of the magnetic properties of such complex subjects. On one hand, the bulk measurements with vibrating sample magnetometer (VSM) could provide more complete information because the VSM signal comes not only from the surface but also from the volume of the massive sample. On the other hand, the information obtained from the whole meander is quite mixed as the shape anisotropy and interactions between different parts of the meanders are crucial. There is no detailed evaluation of local magnetic properties of such structures in the literature and we made a step in this direction. As will be shown below, the proposed methodology seems to be working.

### 2.3. Measurement of MI

**Figure 2:**
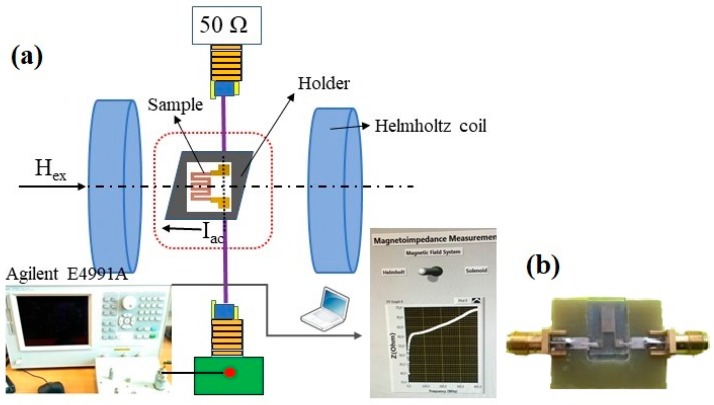
Schematic view of the MI measuring system for meander samples (**a**) and and “microstrype line” type sample holder with incorporated meander sample (**b**).

In order to ensure high-quality MI measurements and to minimize the “microstripe line” holder parasite signal contribution, special sample holders were prepared in accordance with the size of each sample (Figure 2b). The total effective impedance of the sample installed in a measuring circuit (“microstrip” line in this particular case) consisted of two different parts. The first one was the intrinsic impedance of the sample. It changes under the application of the external magnetic field. The second one is the external impedance attributed to the circuit itself. The last contribution does not change in the course of the external magnetic field application. This means that the measured effect is lower than the one corresponding to the intrinsic impedance of the MI sensor element.

The impedance was measured as a function of alternating current frequency in the frequency range of 1 to 200 MHz using an automatic system based on a precision Agilent HP E4991A impedance analyzer. The calibration of the system and mathematical subtraction of the contributions of the test fixture were in accordance with a previously established procedure [29]. Total impedance (Z) was measured as a function of the external magnetic field for the exciting current amplitude of 10 mA in all measurements. The external quasi-static magnetic field (H) generated by Helmholtz coils was changed in the interval +110 to −110 Oe and aligned with the long parts of the meander.

Measurements in the decreasing field were denominated as the “down” branch; measurements in the increasing field were denominated as the “up” branch. The MI ratio for total impedance variation and its real part in the external magnetic field were calculated as follows:

ΔZ/Z = 100 × (Z(H) − Z(H_max_))/Z(H_max_) (1)

ΔR/R = 100 × (R(H) − R(H_max_))/R(H_max_) (2)

where H_max_ = 110 Oe. The maximum ratio at each frequency was denoted ΔZ/Z_max_ for the total impedance and ΔR/R_max_ for its real part. An important characteristic of the MI response is the maximal sensitivity with respect to the external magnetic field. The MI sensitivity with respect to the applied magnetic field was calculated as follows:

S(ΔZ/Z) = Δ(ΔZ/Z)/ΔH(3)

S(ΔR/R) = Δ(ΔR/R)/ΔH(4)

where ΔH ≈ 0.1 Oe is the field increment for calculation of ΔZ and ΔR.

## 3. Results and Discussion

**Figure 3:**
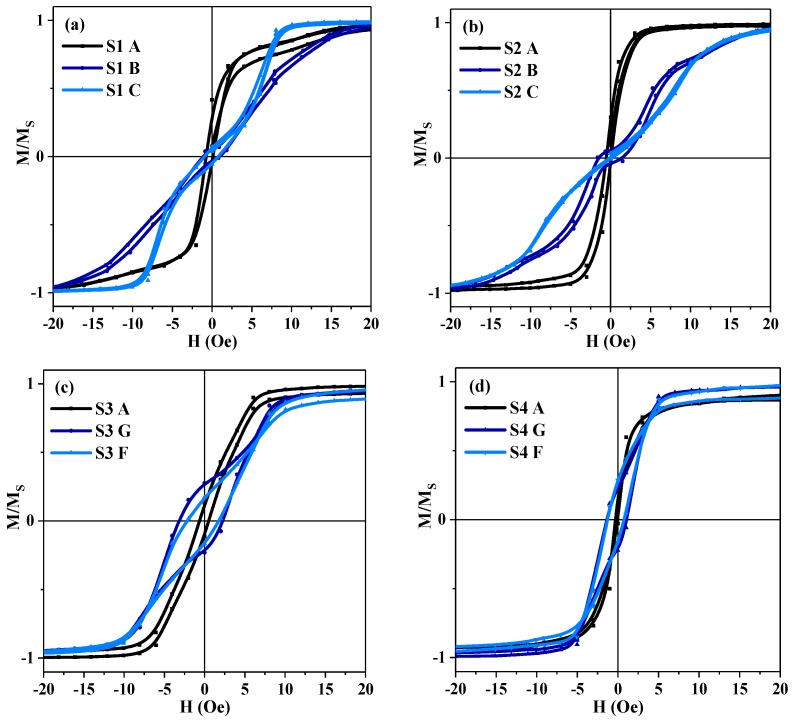
MOKE hysteresis loops taken from the corresponding characteristic sections A, B (G), C (F) of different Co_75_Fe_10_Ni_2_Si_8_B_5_ meander samples. The external magnetic field is applied parallel to the long side of the meanders and perpendicular to the induced magnetic anisotropy axis.

The quasi-static magnetic properties of the meanders were measured by the MOKE microscope. Hysteresis loops taken from the characteristic sections of the samples (Figure 1) are shown in the Figure 3. The hysteresis loops in point A have the small coercive force and high magnetic permeability, which are common for amorphous magnetically soft ribbons [10,29]. More complex types of magnetization reversal were observed for the non-external long sections of the meander due to the complicated process of magnetization interactions with the adjacent parts of the element. Although, one can notice a great difference between the magnetization processes in points B and C of the samples S1, S2 and S3. In the case of the sample S4, only a slight increase in the coercive force takes place. As this sample is longer than the others, one can suppose that the changes in the type of the hysteresis loops are caused by an influence of the smaller contribution of the meanders’ round sections (as points D and H in Figure 2) as well as due to the stronger contribution of the demagnetizing factor of the elongated parts. Although the details of such a mechanism are not clear, the origin can be explained by the presence of the stray fields of different meanders’ areas and surface, including the border defects that appeared during the samples’ production process. At the same time, in order to obtain statistics and better insight, M(H) loops were measured many times at the same position and allow a small displacement. As the M(H) loops in these conditions did not change very much we have concluded that border effects were not crucial.

**Figure 4:**
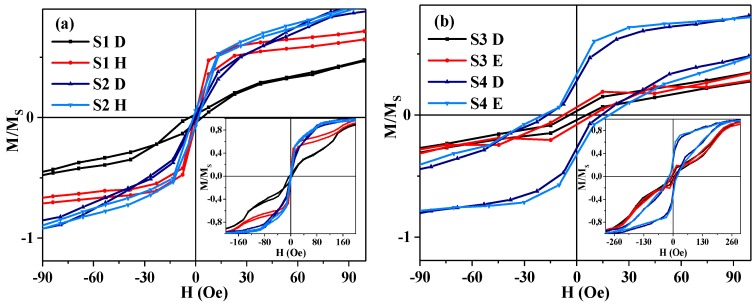
MOKE hysteresis loops taken from the same turning sections of the three-turn (**a**) and six-turn (**b**) Co_75_Fe_10_Ni_2_Si_8_B_5_ meander samples.

Also, one can notice that the magnetic properties of the turning sections of meanders (points D, E, H) differ from the properties of the middle sections (Figure 3 and Figure 4). In these regions the material of the meander reaches a saturated state only in very high magnetic fields above 200 Oe. Moreover, in the case of the sample S4 a certain increase in the coercive force is observed. This fact supports our hypothesis about role of the shape, and to a lesser extent the defects caused by the etching on local magnetic properties and effective magnetic anisotropy axis dispersion.

Although point-by-point analysis of the local hysteresis loops is useful, one can also compare the whole set of the obtained shapes of M(H) loops. It is clear that in the S4 case, M(H) loops for very different points differ from each other to a lesser extent, i.e., the magnetic material shows a lower degree of inhomogeneity in the sense of the dominating magnetization mechanisms. A similar concept was previously discussed in the literature for non-patterned amorphous and nanocrystalline ribbon, we refer to the role of the anisotropy distribution in the formation of the MI response [30,31].

**Figure 5:**
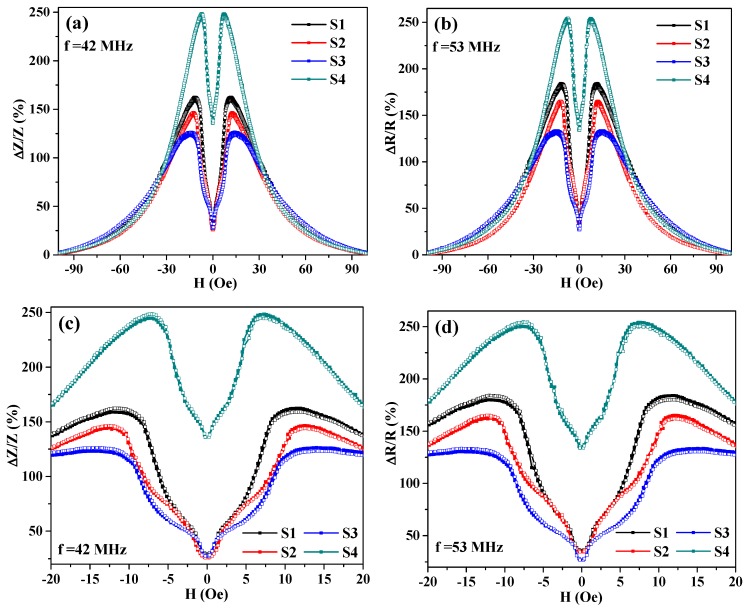
Field dependence of the MI ratio for the total impedance (**a**,**c**) and its real part (**b**,**d**) for the Co_75_Fe_10_Ni_2_Si_8_B_5_ meander samples; (**c**,**d**)—the same dependencies in a small field range. “Down” curves for the magnetic field change from 100 Oe to −100 Oe are marked with solid symbols, “Up” curves for the magnetic field change from −100 Oe to 100 Oe are marked with empty symbols.

**Figure 6:**
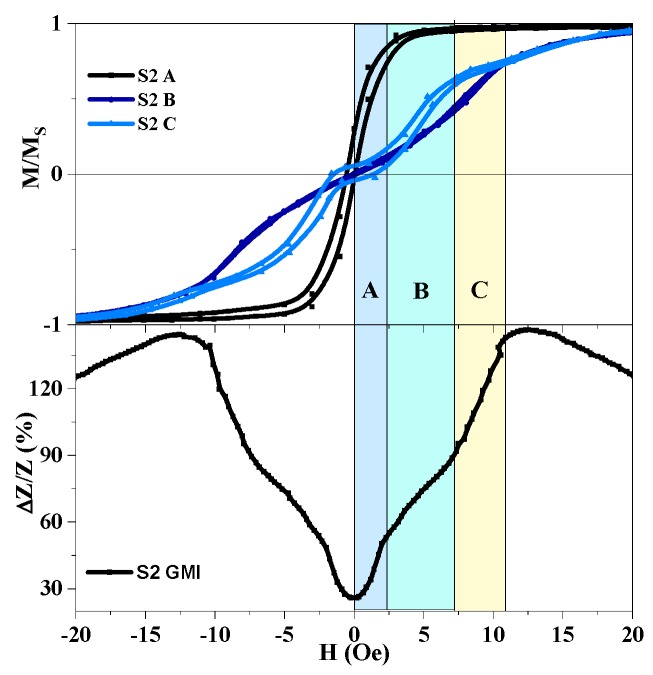
Comparison of the magnetization curves and field dependence of the MI ratio for total impedance of the three-turns Co_75_Fe_10_Ni_2_Si_8_B_5_ meander S2. Characteristic field intervals with different curvature of the GMI curves are marked as A, B and C.

As it can be seen (Figure 5 and Figure 6), complex quasistatic magnetic properties of the meander samples also reflect their MI responses. One can notice several field intervals with different curvature of the MI curves, i.e., behavior, which is usually not observed for simple amorphous ribbon quenched samples [10] in the shape of a single elongated stripe. These intervals are marked as A, B and C (Figure 6) characteristic areas.

In small magnetic fields (interval A) the border parts of the meanders (point A) quickly magnetize, meanwhile the middle sections keep staying almost demagnetized and hence do not contribute significantly to the MI response. In the higher fields (intervals B and C) the border sections become saturated and stop contributing to the MI. The second change of curvature of M(H) curves is observed for both points B and C. Although the explanation of the origin of such behavior requires an additional investigation of the position of about 10 Oe, for both M(H) and MI, the curves indicate a strong contribution of the anisotropy field.

**Figure 7:**
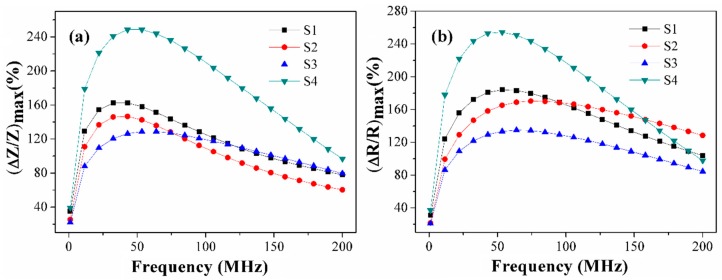
Frequency dependence of the maximal MI ratio for the (**a**) total impedance and (**b**) its real part for Co_75_Fe_10_Ni_2_Si_8_B_5_ meander samples.

**Figure 8:**
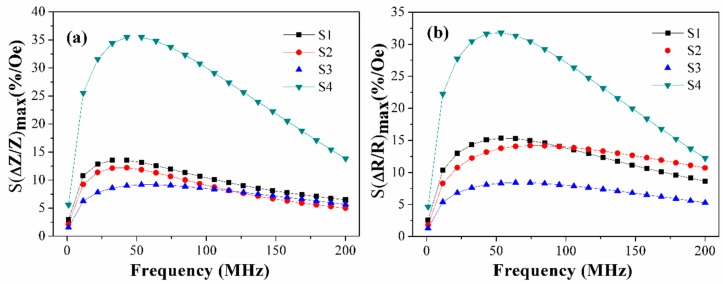
Frequency dependence of the sensitivity of the MI ratio for (**a**) total impedance and (**b**) its real part for Co_75_Fe_10_Ni_2_Si_8_B_5_ meander samples.

Despite the complexity of the dynamic magnetization process described above the meander samples used in this work show high MI response and its sensitivity to an external magnetic field (Figure 7 and Figure 8). As is shown in Figure 8, the S4 meander demonstrates the highest values for both parameters: GMI response ΔZ/Z = 244% and its sensitivity S(ΔZ/Z) = 35%/Oe. It also correlates with Figure 3, which shows that there is only a slight difference in magnetization curves for the different sample areas. As expected, the highest MI effect and its sensitivity are observed for the case where the most uniform anisotropy is insured. Both effects can be explained by a lower anisotropy axis dispersion in comparison with the other samples because of its longer length, and hence a smaller impact of the turning sections or the shape anisotropy.

The frequency dependences of the maximum of the MI ratio for total impedance and the real part of the total impedance are shown in Figure 7. The maximum ΔZ/Z_max_ ratio of about 250% appeared at a frequency of 42 MHz for the six-turn sample S4 (Figure 7a). However, the ΔZ/Z_max_ ratios (162% for S1 and 146% for S2) at a lower frequency of 32 MHz was larger than that of the six-turn sample S3 (129%). The main reason may be that S1 and S2 samples had a better proportion of width and spacing. Compared with the ΔZ/Z_max_ ratios of the three-turn samples S1 and S2, we can find that the MI ratio of the sample S1 with a narrow line-width was larger than that of sample S2. These results were consistent with the earlier reports [26,30].

In the frequency range 100-200 MHz, there is no obvious difference for ΔZ/Z_max_ ratios of samples S1 and S3. Compared with the six-turn meanders, the increase of length had a strong impact on MI enhancement. The maximum ΔR/R_max_ ratios was 254% obtained at a frequency of 53 MHz from the sample S4 (Figure 7b). The tendency for (ΔR/R)_max_ is similar to that of (ΔZ/Z)_max_. However, the ΔR/R_max_ ratios had a conspicuous distinction in the frequency range 1–200 MHz: the working frequencies correspond to the ΔR/R_max_ ratios for samples S1–S4 are higher than that ΔZ/Z_max_ ratios.

Figure 8 shows the frequency dependence of the sensitivity of the MI ratio for total impedance and its real part for different meanders. A general view of the frequency dependence of the MI maximum (both for real part and the total impedance) is quite similar to the (ΔR/R)_max_ (ΔZ/Z)_max_ measured for single stripe amorphous ribbons. The origin of such behavior was previously discussed in the literature. Under conditions of strong impedance variation, the constant external magnetic field and transverse magnetic field created by the alternate current make a contribution to the transverse dynamic permeability. With the frequency increase, the domain walls become affected by stronger eddy-current damping, the anisotropy field gradually increases, magnetic permeability decreases leading to decrease in MI value. More details can be found elsewhere [2,32,33,34].

The sensitivity for the total impedance MI ratio maxima S(ΔZ/Z)_max_ and for its real part maxima S(ΔR/R)_max_ were calculated in the field of 7 Oe, 14 Oe and 12 Oe respectively. The ΔZ/Z_max_ sensitivity of meander samples S4, S3, S2 and S1 were 36%/Oe, 9%/Oe 12%/Oe and 14%/Oe, respectively. These dependences reveal the same tendencies as those found for the frequency dependence the MI ratio maximum for the total impedance and its real part (Figure 8). The highest S(ΔZ/Z)_max_ and S(ΔR/R)_max_ (36%) were observed in the case of the sample S4 with increased frequency. The sensitivity of the MI ratio for total impedance and its real part of sample S3 were median. The values of sensitivity were also adequate for sensor applications from the point of view of magnetic field sensitivity.

**Figure 9:**
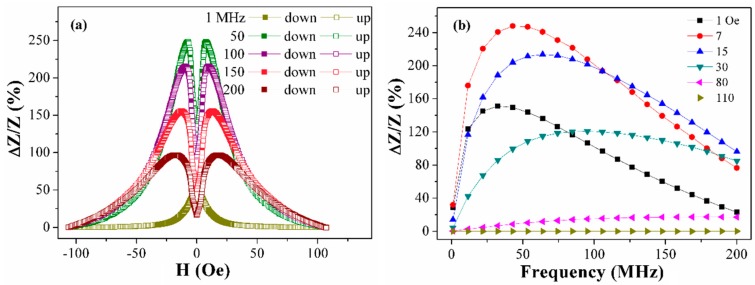
(**a**) Field and (**b**) frequency dependence of the MI ratio of Co_75_Fe_10_Ni_2_Si_8_B_5_ patterned meander element S4.

Table 2 lists the sensitivity of the MI ratio for total impedance and its real part for different samples and the corresponding different magnetic fields and frequencies. The sample S4 had better MI characteristics including a wider range of the fields suitable for application. Therefore, the field and frequency dependence of the MI ratio of the S4 meander were investigated in more detail (Figure 9). Measurements in the decreasing field after magnetic saturation were defined as “down” branch and the measurements in the increasing field after saturation were defined as the “up” branch.

The MI value is almost constant in higher fields due to the saturated sample state. Magnetoimpedance ratio in an external field of 30 Oe also varies slightly in the frequency range 40–200 MHz. A significant change in the MI ratio was observed by varying not only the magnitude of the external magnetic field from 0 to 15 Oe but also the frequency of the exciting current. The optimum mode of MI-detection by the S4 element is frequency range up to 40 MHz in low fields up to 15 Oe. As the biodetection of the magnetic markers requires the highest sensitivity with respect to the applied field, due to the very small value of the stray fields of magnetic nanoparticles employed as biomolecular labels [35], the S4 element seems to be the best candidate for this kind of application.

## 4. Conclusions

In summary, surface magnetic properties and the magnetoimpedance effect of patterned soft ferromagnetic Co_75_Fe_10_Ni_2_Si_8_B_5_ ribbon-based meander sensor elements have been investigated. Samples with a different number of turns, line width and length were fabricated and studied. The results showed that the length of the samples plays the most important role in the formation of well-defined magnetic anisotropy, and hence a higher homogeneity and MI response. The maximum MI ratio of the total impedance of 250% for the exciting current frequency of 45 MHz, and its sensitivity to the external magnetic field of 36%/Oe were observed for the longest six-turn meander sample. These results can be used for further investigations and the production of magnetic field sensors based on the MI effect for the cases focused on large drop analysis.

## Figures and Tables

**Table 1:** Selected parameters of Co_75_Fe_10_Ni_2_Si_8_B_5_ meanders.

Sample	Number of Turns	Spacing	Width	Length
S1	3	50 µm	300 µm	5 mm
S2	3	50 µm	400 µm	5 mm
S3	6	40 µm	250 µm	5 mm
S4	6	40 µm	250 µm	8 mm

**Table 2:** Comparison of sensitivity of the MI ratio for total impedance and its real part for Co_75_Fe_10_Ni_2_Si_8_B_5_ meander samples, and the corresponding different magnetic fields and frequencies.

Sample	S (ΔZ/Z)(%/Oe)	H (Oe)	f (MHz)	S (ΔR/R)(%/Oe)	H (Oe)	f (MHz)
S1	14%	12	32	15	12	53
S2	12%	12	42	14	12	74
S3	9%	14	63	10	14	63
S4	36%	7	42	36	7	53
